# Hormonal Regulation of Physiology, Innate Immunity and Antibody Response to H1N1 Influenza Virus Infection During Pregnancy

**DOI:** 10.3389/fimmu.2018.02455

**Published:** 2018-10-29

**Authors:** Elizabeth Q. Littauer, Ioanna Skountzou

**Affiliations:** ^1^Emory Vaccine Center, Emory University School of Medicine, Atlanta, GA, United States; ^2^Department of Microbiology and Immunology, Emory University School of Medicine, Atlanta, GA, United States

**Keywords:** pregnancy, H1N1 influenza virus infection, animal models, sex hormones, maternal immune response

## Abstract

In 2009, the H1N1 swine flu pandemic highlighted the vulnerability of pregnant women to influenza viral infection. Pregnant women infected with influenza A virus were at increased risk of hospitalization and severe acute respiratory distress syndrome (ARDS), which is associated with high mortality, while their newborns had an increased risk of pre-term birth or low birth weight. Pregnant women have a unique immunological profile modulated by the sex hormones required to maintain pregnancy, namely progesterone and estrogens. The role of these hormones in coordinating maternal immunotolerance in uterine tissue and cellular subsets has been well researched; however, these hormones have wide-ranging effects outside the uterus in modulating the immune response to disease. In this review, we compile research findings in the clinic and in animal models that elaborate on the unique features of H1N1 influenza A viral pathogenesis during pregnancy, the crosstalk between innate immune signaling and hormonal regulation during pregnancy, and the role of pregnancy hormones in modulating cellular responses to influenza A viral infection at mid-gestation. We highlight the ways in which lung architecture and function is stressed by pregnancy, increasing baseline inflammation prior to infection. We demonstrate that infection disrupts progesterone production and upregulates inflammatory mediators, such as cyclooxygenase-2 (COX-2) and prostaglandins, resulting in pre-term labor and spontaneous abortions. Lastly, we profile the ways in which pregnancy alters innate and adaptive cellular immune responses to H1N1 influenza viral infection, and the ways in which these protect fetal development at the expense of effective long-term immune memory. Thus, we highlight advancements in the field of reproductive immunology in response to viral infection and illustrate how that knowledge might be used to develop more effective post-infection therapies and vaccination strategies.

## Introduction

Influenza viruses are segmented, negative-stranded enveloped RNA viruses that cause respiratory infections, fever, malaise, coughing, and mucus production. Influenza viruses are divided into A, B, C, and D types; while all A, B, and C can be infectious in humans with influenza A viruses (IAVs) causing the most widespread disease, influenza D virus is not known to infection humans (1, 2). Influenza A viruses are further classified by the antigenicity of their surface proteins hemagglutinin (HA) and neuraminidase (NA), which are encoded on individual segments of viral RNA, and define the host range of each virus (1, 3). Through a process unique to influenza and other segmented genome viruses, co-infection of different viral subtypes in human, swine, avian or other animal hosts can result in reassortment leading to antigenically unique novel viruses that may take advantage of an immunologically naïve host species (4). This process of reassortment resulted in the emergence of the four major influenza virus strains causing pandemics; the 1918 H1N1 Spanish influenza, the 1957 H2N2 Asian influenza, the 1968 H3N2 Hong Kong influenza, and the 2009 H1N1/09 swine influenza, that infected up to 50% of the global population and caused a significant increase in mortality (2, 3). While infection can occur year-round, the epidemiology of influenza virus infection is seasonal, causing peak illness in November through March in the Northern Hemisphere and approximately 200,000 hospitalizations and 36,000 deaths annually in the United States (3). Currently, the most common circulating influenza A subtypes are H1N1 and H3N2, which are included in quadrivalent vaccines together with the type B influenza lineages Yamagata and Victoria (5). Due to the wide variety of circulating viruses and the frequency of genetic reassortment between subtypes, vaccination is required annually to provide immune protection during each influenza season although it may not be complete if there is mismatch between predicted strains included in the vaccine and the resultant circulating strains in the following season.

Seasonal influenza infections during the second and third trimester of gestation increase the morbidity of pregnant women with higher hospitalization rates than the general population and mortality (6). Pregnant women have been particularly vulnerable to pandemic influenza viruses showing up to 45% increased morbidity and mortality, and they were at an increased risk of higher cardiopulmonary complications and gestational abnormalities during the four major influenza pandemics in the past 100 years (7–9). While pregnant women typically represent 1% of the American population, during the 2009 H1N1 pandemic they comprised 6.4% of all hospitalizations and 4.3% of all deaths (10, 11). Over half of those women hospitalized for H1N1 influenza virus infection had another pre-existing condition, such as asthma, high blood pressure, and diabetes; women with asthma represented 43.5% of deaths from influenza virus infection during pregnancy, which is part of a larger phenomenon of enhanced viral pathogenesis and severe outcomes among asthmatic adults (11, 12).

Influenza infection-related complications in fetuses and neonates have been associated with increased risk of miscarriage, pre-term birth, stillbirth, neonatal death, and low birth weight (6, 9, 13, 14). Clinical reports of influenza-like illness (ILI) during pregnancy have been correlated with a five-fold increase in perinatal morbidity and mortality (15). The incidence of pre-term birth increased from 7 per 1,000 births to 39 per 1,000 births and the incidence of stillbirth from 6 to 27 stillbirths per 1,000 births in the 2009 pandemic (9, 10, 16). Specifically, pregnant women who tested positive for the 2009 swine-origin H1N1 virus were more likely to deliver low birthweight infants than pregnant women who delivered following ILI that was not caused by the pandemic strain (17). There is historical evidence that the 1918 and 1957 pandemics produced similar clinical outcomes for pregnant women; however, modern diagnostic procedures employed during the 2009 H1N1 pandemic allow for more direct linkage between influenza viral infection of pregnant mothers and poor outcomes for maternal and neonatal health (9).

The mortality rates reported for both women and their newborns led to initiatives by the Centers for Disease Control and Prevention (CDC) and the World Health Organization (WHO) to increase influenza vaccination coverage in pregnant women (5, 18). Maternal vaccination during the second or third trimester of pregnancy with seasonal trivalent influenza vaccine substantially reduced the incidence of ILI in both mothers and their newborns (19–21) and has not been associated with pre-term delivery or an increase of adverse outcomes for mothers or their infants. In a review of 7 clinical studies, Bratton et al concluded that maternal vaccination reduced the likelihood of stillbirth compared to unvaccinated pregnant mothers (22–24). However, while the conventional intramuscular vaccination has been determined to be reasonably safe for routine use during pregnancy, promoting the transplacental transfer of anti-influenza virus antibodies from mother to fetus, clinical data is inconclusive regarding the efficiency of immune responses to the vaccine when compared to those induced in non-pregnant women (18, 19, 25–27).

Vaccination is widely recommended during pregnancy for the benefit of mother and child; however, vaccination coverage among pregnant women in the United States remains around 50% (6). Research into the specific mechanisms by which H1N1 influenza virus infection causes pregnancy complications and how pregnancy hormones modulate the immune response to infection and vaccination may reveal improved routes of therapy for women infected with influenza A virus during pregnancy. This review will discuss how H1N1 influenza virus infection disrupts maternal lung and placental function as well as the role of pregnancy hormones in shaping the innate and cellular immune responses to H1N1 influenza virus infection.

### The physiology of influenza A virus infection and immune responses

Human influenza A virus is typically transmitted through respiratory droplets and inhaled into the nasopharynx. Initial virus infection occurs when hemagglutinin (HA), a surface protein on influenza virions, binds to α2,6-linked sialic acids that are widely expressed on the surface of ciliated airway epithelial cells throughout the upper respiratory tract (28, 29). Viruses are then endocytosed primarily via a clathrin-mediated pathway; upon acidification of the vesicle containing influenza virions, the HA protein is triggered to fuse viral and cellular membranes, releasing the viral genome into the cell (30–32). The eight negative-stranded RNA segments of the viral genome are then translocated to the nucleus, where they replicate using the associated viral RNA-dependent RNA polymerase through a complementary RNA (cRNA) replication intermediate (33). In parallel, viral transcripts are generated using stretches of capped cellular RNA molecules as primers (“cap-snatching”). After translation of the viral transcripts in the cytoplasm, the eight genome segments are packaged into virions which are released from the apical cellular membranes to infect nearby cells and thus result in viral amplification (32, 34). Viruses shed from the nasopharynx may be inhaled further into the lower respiratory tract causing severe pulmonary infections or transmitted through respiratory droplets from the upper respiratory compartment to the next person. People infected with 2009 H1N1 influenza A virus were contagious as early as 12 hours post-inoculation and typically began experiencing symptoms approximately 2 days following infection (35).

The release of viral RNA within the cell activates innate immune signaling pathways, especially toll-like receptors (TLRs) and retinoic-acid-inducible gene 1 (RIG1), which induce the expression of the pro-inflammatory cytokines interferon-α (IFN-α) and interferon-β (IFN-β) (36, 37). The secretion of these cytokines activates the surrounding epithelial cells to express antiviral genes that hamper viral entry and replication and recruit innate immune cells to the site of infection (37–39). Natural killer (NK) cells and neutrophils kill infected cells, and additional cytokines expressed by activated airway epithelium and innate immune cells induce fever and mucus production, which in turn results in coughing and rhinorrhea to shed the virus and cellular debris from the lungs and the nasopharynx (38, 40). These infection-induced responses are the hallmark of influenza illness symptomatology, and in clinically vulnerable populations, chest congestion due to viral infection and the ensuing immune response can lead to bronchitis, pneumonia, and secondary bacterial infections (41–43). Viral clearance finally occurs around 8 to 10 days after the onset of symptoms when the adaptive immune response mounts virus-specific clearing of infected tissue (38, 44). Viral antigen is taken up and processed by dendritic cells which migrate upon activation to draining mediastinal lymph nodes and prime naïve resident T cells to respond to the infection (45). Humoral memory is developed when naïve B cells are primed by soluble antigen and costimulated by CD4+ T follicular helper (TFH) cells to mature into plasmablasts that will then traffic to the site of the infection and secrete virus-specific neutralizing antibodies (46). Ultimately, following costimulatory help from CD4+ T cells, CD8+ cytotoxic T lymphocytes will traffic to the lungs and eradicate virus-infected cells (38, 46, 47). Upon resolution of disease, alveolar macrophages clear cellular debris, and basal stem cells regenerate airway epithelium to restore healthy tissue (48, 49). In animal models for influenza viral pathogenesis, it was demonstrated that virus-specific CD103^+^ CD8^+^ tissue resident memory (TRM) T cells in the lungs could provide rapid response upon the next infection and memory B cells persisted in the mediastinal lymph nodes to secrete virus-neutralizing antibody into circulation upon restimulation (50–52).

Research into the specific mechanisms of H1N1 influenza A virus binding, entry, RNA replication, transmission, and induction of the host immune system has been extensive since the 1918 Spanish influenza pandemic; however, investigations into how these mechanisms manifest in disease in immunologically unique populations, such as infants, the elderly, HIV^+^ or asthmatic patients, and pregnant women have been limited.

### Hormonal regulation of pregnancy and immune signaling are delicately balanced to protect fetal development

Female reproduction is regulated predominately via estrogen, progesterone, luteinizing hormone (LH), and follicular stimulating hormone (FSH). Estrogen receptors (ERs) and progesterone receptors (PGRs) are typically expressed within the cytosol and translocated to the nucleus upon ligand binding to induce a suite of genes encoding immunomodulators, regulators for tissue remodeling, mammary gland development, metabolism, lung physiology and function (53–55). LH and FSH are synthesized in the anterior pituitary gland and coordinate the decidualization of the uterine endometrium as well as the release of oocytes from mature ovarian follicles into the uterus for fertilization (56). A fertilized oocyte develops into a blastocyst, and then the outer layer of the blastocyst forms a polarized structure called the trophectoderm (57). The trophoctoderm layer implants in the uterine wall to become syncytiotrophoblasts that secrete human chorionic gonadotropin (hCG) and develop into fetal placental chorionic villi (57). Placental hCG expression signals the maternal corpus luteum to produce progesterone, which maintains the appropriate thickness and vascularization of the endometrium to support embryonic growth (56).

Insufficient progesterone production has been associated with infertility and recurrent spontaneous abortions, indicating that variations in progesterone levels as a result of infectious disease are not well tolerated by maternal physiology and may result in miscarriage or pre-term birth (58–60). Sex hormones play a crucial role in organizing endometrial granulated lymphocytes (EGLs) in the innermost layer of epithelial tissue in the uterus and populations of uterine natural killer (NK) cells, dendritic cells, macrophages, and memory and regulatory T cells are tightly controlled throughout the first, second, and third trimesters of pregnancy (56, 61, 62). Estrogens are expressed in several major forms, mainly estradiol (E2) and estriol (E3); each can have biphasic effects in stimulating pro-inflammatory signaling via mitogen-associated protein kinases (MAPKs) and NK activation at low concentrations or enhancing the expression of PD-L1 on T cells and the synthesis of TGF- β and IL-10 at high concentrations (62). Progesterone receptors are expressed broadly on most immune cell subsets and are produced in higher levels in females (62–64). In the uterus, progesterone induces the transition of naïve Th0 cells into IL-4, IL-5, and IL-6 secreting Th2 memory cells upon antigen recognition; these Th2 cells are critical for coordinating immune tolerant cytokine crosstalk between the maternal and fetal sides of the placenta and preventing intrauterine NK cell activation against fetal trophoblasts (61). The expression of IL-4 and IL-6 then promotes hCG secretion from the corpus luteum, which in turn releases more progesterone, creating a positive feedback loop for the amplification of hormone-mediated Th2 polarization (61). This phenomenon has been shown to be important for maintaining immune tolerance, and recurrent miscarriage is associated with a predominance of Th1 memory cells in the endometrium (65). Estrogens have also been implicated in inducing CD4^+^ CD25^+^ T regulatory cells (Tregs) and are critical for maintaining tolerance within the maternal-fetal interface (66). Progesterone also upregulates the activity of uterine Tregs, which act as suppressors of inflammatory immune subsets, particularly NK cells and macrophages resident to the endometrium (62, 64). In this way, estrogens and progesterone coordinate an environment in which both uterine epithelial cells and innate immune cells resident to uterine tissue will tolerate the implantation of a fertilized oocyte and the development of a placenta and fetus.

The structure and cellular composition of placenta is critical to maintaining fetal growth and development as well as protection from inflammation. Fetal placenta develops from the cells in the implanted blastocyst as it transitions into the trophoblast which differentiates into cytotrophoblasts and synciotrophoblasts. Both cell types contribute to the development of chorionic villi that form an interface with the uterine decidua (57). Here, maternal blood makes direct contact with fetal cells, allowing for gas, nutrient, and waste exchange but also providing a potential door for entry of bacteria, viruses, and parasites to a fetus with an undeveloped immune system (67). Few pathogens can cross the placental barrier from the mother to the fetus. TORCH pathogens [*Toxoplasma gondii*; other pathogens including, human immunodeficiency virus (HIV), varicella zoster virus (VZV), malaria-causing *Plasmodium* species, *Listeria monocytogenes, Treponema pallidum*, parvoviruses B19, enteroviruses, and recently, Zika virus; *r*ubella virus; *c*ytomegalovirus (CMV); and *h*erpes simplex virus 1 and 2 (HSV)] are associated with fetal and neonatal morbidity and mortality from CNS abnormalities, microcephaly, blindness, deafness, premature birth or low birth weight (67, 68). However, there is limited evidence that influenza A virus crosses the maternal-fetal barrier. Despite the demonstrated ability of the 2009 pandemic strain to infect fetal trophoblasts, the development of chorionic villitis and the widespread reports of increased risk of maternal and fetal mortality, there were few conclusive cases of vertical transmission via the placenta (69, 70). Thus, poor fetal outcomes during pregnancy are likely due to indirect exposure to maternal inflammatory cytokine expression and dysregulation of pregnancy-supportive hormones.

In addition to preventing pathogen entry into the fetal bloodstream, it is also critical that cytokines that make it across the placental syncytiotrophoblast layer into the fetal circulatory system do not cause inflammation or immune cell activation that interrupts fetal growth and development (71, 72). Clinical reports of maternal inflammation and infection during pregnancy have been associated, although inconclusively, with the development of autism, bipolar disorders, and schizophrenia in children born to mothers infected with influenza A virus during pregnancy (73–75). Peripheral blood mononuclear cells (PBMCs) isolated from healthy pregnant women and co-cultured with 2009 pandemic influenza A virus subtype H1N1 or circulating rhinovirus strains (HRV43 and HRV1B) had significantly reduced IFN-α and IFN-γ responses, indicating increased susceptibility to severe outcomes of viral infection during pregnancy (76, 77) A shift away from inflammatory Th1 cytokines (TNF-α, IFN-γ, IL-2) can limit potential cytotoxic damage to the fetus and placenta (61, 62). Sex hormones coordinate this shift by activating transcriptional factors via transmembrane and intracellular receptors which activate a suite of anti-abortive, pro-pregnancy genes (63). For example, progesterone activates progesterone-induced binding factor (PIBF) in lymphocytes, which in turn promotes the synthesis of IL-3, IL-4, and IL-10, while reducing the expression of IL-12 (78, 79). PIBF also inhibits NK cell degranulation, and decreased PIBF expression is linked to recurrent spontaneous abortions (79, 80). Thus, hormone-mediated suppression of inflammatory cytokine production and cellular activation is critical to successful pregnancy in the short-term by protecting the placenta from inflammation that could trigger pre-term birth or neurodevelopment damage; however, proper inflammatory signals must still be activated to recruit innate immune cells and CD8^+^ T cells in order to clear virus-infected tissue.

While pre-term birth and low birth weight neonates have been well-documented outcomes of the 2009 H1N1 influenza virus infection in pregnant women, a mechanism for this phenotype is unclear, though placental transmission of inflammatory cytokines, dysregulated hormone signaling, and oxygen deprivation due to maternal respiratory distress have all been implicated (62, 81, 82). The effect of the hormonal millieu during pregnancy on innate immune responses is complicated, and *ex vivo* modeling of a single subset of cells may not depict the entire story of hormonal, cytokine and immune cell signaling between lung, fetus, and placenta in an infected pregnant woman. Clinical samples from pregnant women are limited to blood, post-partum placenta, and post-mortem tissues, leaving research questions about maternal lung function and immune responses to non-fatal influenza viral infection unanswered.

Rodent models, particularly mice, are a commonly accepted experimental tool for preclinical research studies due to their hemochorial placental structures, recapitulation of influenza viral pathogenesis seen in humans, and their cost effectiveness over multiple time points (29). One approach for the elucidation of these mechanisms is to expose healthy non-pregnant female mice to low doses of sex hormones comparable to birth control or high doses comparable to those of pregnancy. Pazos et al. implanted female C57BL/6 mice with degradable 17β-estradiol (E2 in mice) pellets to yield serum E2 levels of third trimester pregnancy and infected them with H1N1 PR8 virus; mice implanted with E2 exhibited reduced type I IFN signaling and impaired CD8^+^ T cell function compared to infected non-implanted female mice (83). Robinson et al proposed that 17β-estradiol has protective effect during pregnancy; ovariectomized and E2-implanted female C57BL/6 mice infected with H1N1 PR8 influenza virus exhibited enhanced recruitment of neutrophils and virus-specific T cells, which promote viral clearance (84). In contrast, studies involving pregnant mice demonstrated that while individual expression of estrogen or progesterone may limit inflammation, the condition of pregnancy resulted in elevated inflammatory responses to influenza virus infection compared to the immune responses of infected non-pregnant female mice (85–87). Pregnant mice infected with a mouse-adapted, 2009 H1N1 influenza virus expressed elevated levels of IL-1α, IL-6, granulocyte-colony stimulating factor (G-CSF), monocyte chemotactic protein (MCP-1), CXCL1, and RANTES and experienced more severe pathology and mortality when compared to non-pregnant mice (88). These cytokines were highly expressed in humans who died as a result of 2009 H1N1 influenza A virus (87, 89). These differences in immune responses between hormone-treated mice and pregnant mice infected with influenza virus highlights how immune and endocrine crosstalk between mother, fetus, and placenta has far-reaching consequences beyond classical reproductive tissues and complicates our understanding of typical H1N1 viral pathogenesis.

The genetic background of mouse strain is also significant in the selection of a pregnant mouse model. C57BL/6 mice classically tend toward Th1-type immune responses while mice with BALB/c genetic backgrounds tend toward Th2-type immune responses (90, 91). Differences in genetic background have been shown to cause variability in viral pathogenesis, inflammatory cytokine response, pulmonary microRNA expression, alveolar macrophage viability following intranasal infection with 2009 H1N1 pandemic influenza virus strains (92–94). Strain differences also affect the physiological response to influenza viral infection during pregnancy. Recent findings in C57BL/6 mice have highlighted that pregnancy significantly enhances lung function by increasing respiratory compliance and total lung capacity and that influenza virus infection does not alter lung tidal volume, minute ventilation, diffusing capacity, and compliance as shown in non-pregnant infected mice. The authors observed less inflammation in the lungs of infected pregnant mice and suggested that this is a protective mechanism against maternal respiratory damage during pregnancy (95). However, we and others have shown in the BALB/c mouse model that pregnancy increases lung inflammation and expression of stress-induced prostaglandins (PGs) and cyclooxygenase-2 (COX-2) prior to infection and that IAV infection enhances immunopathology in the lungs of pregnant mice relative to non-pregnant mice (86–88). Oxidative stress interferes with lipid raft clustering and has been shown to inhibit the ability of PIBF to bind its transmembrane receptor and IL-4R to induce the STAT6 signaling pathway; this interference reduces the sensitivity of cells to PIBF (96, 97). Thus, influenza viral infection and subsequent oxidative stress may interfere with the unique lung and mucosal physiology tightly regulated by sex hormones toward successful pregnancy and fetal development.

### Humoral immune responses following infection and vaccination during pregnancy

The natural outcome of infection is the development of immunological memory to prevent re-infection and future cellular damage. As discussed previously, soluble viral antigen released from infected cells in the lungs primes naive B cells in the proximal draining lymph nodes by binding to the B cell receptor (BCR), crosslinking several BCRs in the process and amplifying an activation signal (46, 98–100). Additional costimulation by CD4^+^ helper T cells responding to processed viral antigen in MHC class II proteins on the B cell's surface is required to fully activate B cells and provides a second activation signal, resulting in clonal proliferation and amplification of antibodies specific for influenza viral antigens (46). Selection for B cells with BCRs with highest affinity for the viral antigen occurs in the germinal centers found in secondary lymphoid tissues such as the spleen. In the latter, cells undergo somatic hypermutation, a process by which DNA encoding hypervariable Ig regions is broken by activation-induced deaminase (AID) and uracil-DNA glycosylase (UNG) and repaired by MSH2/6 and REV1. The accumulating mutations may result in the generation of antibodies with an increased affinity to viral antigens (101, 102). Immunoglobulin class switching increases the range of functions by recombining antibody variable regions encoding specificity for influenza viral proteins with constant regions encoding receptors for various innate immune cells and intercellular trafficking (102). Ultimately, most antibody-secreting cells (ASCs) will undergo apoptosis following viral clearance. Only a small percentage of these high-specificity B cell clones will become plasma cells that secrete low levels of antibody into the serum for months, or memory cells that reside in the bone marrow, and can be reactivated to provide antibody responses to a subsequent infection (46, 103).

This system-wide coordination of B cell activation and survival in response to foreign antigen delicately balances the pregnant mother's serum antibody levels to both provide the benefits of transplacental immunity to the fetus and avoid the development of fetal-reactive antibodies. The competing priorities of fetal antigen tolerance and the production of antibodies that can be transplacentally conferred to the fetus to promote neonatal immunity are tightly regulated by pregnancy hormones. Clinical evidence has long documented that the symptoms of autoimmune diseases arising from the generation of antibodies against self-antigen tend to recede during pregnancy and resurge after parturition and breastfeeding, indicating that pregnancy hormones play a role in coordinating immune tolerance at the local uterine and systemic level (62, 64). The development of autoimmune disorders such as multiple sclerosis, rheumatoid arthritis, and systemic lupus erythematosus (SLE), which are more prevalent in women, have been linked to the effects of estrogen on B cell activation and function (104). Estradiol (E2) has been shown to upregulate Bcl-2, inducing survival of autoreactive B cells and changing signaling thresholds required to induce apoptosis (105, 106). In contrast, progesterone has been established as negative regulator of B cell lymphopoiesis (107–109). Reduced expansion of B cells within a pregnant mother may help establish allotolerance to the fetus by preventing antibody recognition of fetal antigen, which might result in inflammation, lymphocyte cytotoxicity, and complement activation (63). Healthy pregnancy has been shown to suppress B cell lymphopoiesis in BALB/c mice, which could be reversed by the exogenous addition of IL-7 (107, 110, 111). These data suggest that pregnancy may reduce or redirect activated B cells during their migration to the lungs or bone marrow. Differential recruitment of IgA^+^ plasmablasts to the murine mammary glands after parturition and during nursing has been demonstrated, but specific homing receptors have not been identified, suggesting a role of local chemoattractants such as E-selectin (112, 113). In this way, maintaining immunological tolerance to fetal antigen that reaches the maternal circulatory system may require that B cell activation be altered in order to prevent the proliferation of anti-fetal antibodies.

Understanding how pregnancy impacts the development of immune memory is of clinical significance. Immunization has been reported to reduce hospitalization and ILI of pregnant women and their newborns during the flu season with no record of increased adverse events due to vaccination between this group and the unvaccinated population (21, 23, 114, 115). Clinical trials of seasonal trivalent inactivated influenza vaccination (TIV) in Bangladesh showed improved transplacental transfer of influenza-specific antibodies from mother to child (116). However, there are mixed results in how pregnancy affects humoral immunity following vaccination. Schlaudecker et al. reported that pregnant women seroconverted at the same rate as non-pregnant women following TIV but generated lower geometric mean titers (GMTs) against H1N1 (A/California) and H3N2 (A/Perth) viruses (26). Serological analysis from a cohort of influenza A virus (IAV) vaccinated healthy pregnant and non-pregnant women in California showed similar seroconversion rates and numbers of plasmablasts (18). Thus, while influenza vaccination during pregnancy has been demonstrated to be safe and to reduce the incidence of influenza-induced hospitalization and pre-term birth, further research into antibody functionality and expression is still needed.

Pregnancy hormones may coordinate the down-regulation of class-switching or post-translational modifications (i.e., glycosylation, fucosylation, sialylation, etc.) of the antigen-binding (Fab) or receptor binding (Fc) regions of antibodies in order to attenuate potentially inflammatory or anti-fetal immune responses. There have been reports that pregnant women infected with H1N1 pandemic virus in Shenyang, China in 2009 produced an imbalanced proportion of anti-H1N1 IgG1, IgG2, IgG3, and IgG4 antibody subtypes compared with infected non-pregnant women in the same hospital (117). Interestingly, IgG1 is preferentially transported from maternal circulation across the placenta compared to other IgG classes, especially IgG2, although this phenomenon has not been directly linked to influenza infection and vaccination (118–120). While preferential transport from mother to fetus is linked to neonatal Fc receptor (FcRn) expression on placental syncytiotrophoblasts, how pregnancy shifts expression from virus-specific IgG2 to IgG1 requires further investigation.

Antibody isotype classes and generation of specificity are governed by the class-switching of Ig genes and somatic hypermutation of their variable chain-encoding regions (102, 121). Variability is induced primarily by activation-induced cytidine deaminase (AID) and uracil-DNA glycosylase (UNG) that selectively damage DNA and repair it randomly (102, 121, 122). B cells that have complementarity-determining regions (CDRs) that bind best to antigen are selected for by T follicular helper cells (Tfhs) and clonally amplified to flood the circulatory system with virus neutralizing antibodies (123, 124). Estrogen and progesterone seem to work in opposition to each other on the regulation of AID: estrogen receptors bind the *HoxC4* promoter to induce AID activation, while progesterone receptors can bind directly to the AID promoter to inhibit activation (125–127). Glucocorticoids have also been described as negative regulators of AID activation (128). These phenomena are typically described in the context of autoimmune disease regulation and have not been described in the multi-hormonal environment of pregnancy.

Asymmetric glycosylation, or the single glycosylation of one side rather than both sides of the Fab or Fc antibody chains, can result in fine-tuned interactions with antigen and Fc receptors, and these binding affinities are important for antibody-dependent cellular cytotoxicity (ADCC) (129, 130). Pregnancy has been shown to increase the serum and placental concentrations of asymmetrically glycosylated IgG and may provide an explanation for the reduced avidity and virus-binding capability following viral infection during pregnancy (131, 132). Human and murine placental expression of IL-6 has been shown to induce asymmetrical glycosylation of IgG from hybridomas (133, 134). Trophoblast-produced asymmetric antibodies have been documented throughout the placenta (132, 135). However, whether these signaling effects can extend outside the uterus has yet to be determined and would be a major finding in maternal immunity. By reducing binding specificity for antibody effector cells via asymmetric glycosylation, the maternal immune system may be able to mitigate the negative effects of any anti-fetal antibodies that may have developed while still maintaining a population of semi-functional or selectively-functional antibodies that can neutralize pathogens and non-self-entities (131).

## Conclusions

Influenza viral illness causes significant socioeconomic and clinical burden each year (136). While most research focused on the consequences of influenza A (H1N1) virus infection during pregnancy, there is evidence that influenza B virus can also cause significant maternal and fetal complications following mid-gestation infection (137, 138). It remains unclear if seasonal type A (H3N2) virus infection during pregnancy causes similar poor clinical outcomes compared to the severity of complications following type A (H1N1) or type B virus infection during pregnancy (138–141). Lastly, the recently identified highly neurotropic avian H7N9 and H5N1 influenza A reassortants, which could potentially cause pandemics, have been shown to cause severe disease during pregnancy (142–147). The knowledge gained through research of the 2009 pandemic swine-derived influenza A (H1N1) virus may provide the clinical and research community with an improved capacity for the early detection of a novel pandemic virus entering a naïve pregnant population. These studies we have reviewed demonstrate the vulnerability of pregnant women to infectious diseases and the fact that neonatal health is directly dependent on maternal health, doubles the significance of research that results in improved therapies and treatment strategies.

Respiratory infection during pregnancy is of broad interest. While influenza A virus has generated some of the highest morbidity rates following maternal infection, coronavirus outbreaks have also been associated with similar outcomes in mothers and neonates following mid-gestation infection. Infection with severe acute respiratory syndrome (SARS) coronavirus and Middle Eastern respiratory syndrome (MERS) coronavirus have been associated with spontaneous abortion, fetal growth retardation, and maternal and neonatal mortality (148–150). Mid-gestation infection with respiratory syncytial virus (RSV) has been described in rare severe adult cases and has also been associated with pre-term birth and low birth weight in a cohort in Nepal (151, 152). High rates of mortality among infants and toddlers infected with RSV highlights the need for improved understanding of maternal immunity to RSV infection and vaccination during pregnancy, and there is hope that vaccination of mothers during pregnancy can provide passive immunity that will protect the fetus for months after birth (153). None of the previously mentioned viruses were transmitted transplacentally to fetuses, and yet respiratory infection during pregnancy induced significant maternal illness, pre-term labor, low birth weight, or spontaneous abortion.

Early antiviral therapy following H1N1 influenza A virus infection during pregnancy has been shown to significantly reduce pre-term birth, hospitalization in intensive care units (ICUs), and maternal death (11, 154). Seasonal H1N1 influenza A virus induced increased levels of cyclooxygenase-2 (COX-2) and prostaglandin-F2α in the lungs and placenta, providing a mechanism for lung immunopathology and pre-term labor in pregnant mice (88). The anti-inflammatory potential of COX-2 inhibitor therapy has already been proposed for decreasing disease severity caused by the highly pathogenic avian influenza strains H5N1 and H7N9 (155, 156). In addition, COX inhibitor treatment has demonstrated to attenuate the lung expression of granulocyte colony-stimulating factor (G-CSF) and keratinocyte-derived (KC) cytokines elevated in pregnant mice infected by H1N1 A/Brisbane/59/2007 and H1N1 A/California/07/2009 (157, 158). However, while non-steroidal anti-inflammatory drugs (NSAIDs) have been shown to be safe during pregnancy, COX-2 specific inhibitors may induce pre-term labor and musculoskeletal defects (159–161). Viral load was negatively associated with progesterone concentration, and reduced progesterone expression was correlated with pre-term labor in influenza virus-infected pregnant mice (88). Administration of progesterone to female mice following influenza A(H1N1) virus infection reduced immunopathological changes and improved lung epithelial cell regeneration, although it did not reduce viral load (162, 163). Hence, limiting viral replication should be one of many aims for anti-influenza therapy during pregnancy, including the limiting of immunopathology caused by cytokine dysregulation and promoting the healing of damaged airway epithelium following viral clearance.

The connections between viral pathogenesis and reproductive endocrinology makes the field of infectious disease in pregnant women complicated, exciting, and clinically significant. Investigations into the immunological components of infertility, recurrent miscarriage, and preeclampsia have yielded a wealth of information regarding the requirements of immune tolerance and rejection, and this information can provide a platform for understanding healthy pregnancy and how inflammation and hormonal dysregulation will impact maternal health and fetal development. Development of accurate animal pregnancy models across a range of species in coordination with broader clinical sampling from influenza-infected or -vaccinated pregnant women will provide an effective platform for validation of experimental studies and improved therapeutics and treatment for pregnant women and their offspring.

## Author contributions

All authors listed have made a substantial, direct and intellectual contribution to the work, and approved it for publication.

### Conflict of interest statement

The authors declare that the research was conducted in the absence of any commercial or financial relationships that could be construed as a potential conflict of interest.
